# Structural Studies of β-Carbonic Anhydrase from the Green Alga *Coccomyxa*: Inhibitor Complexes with Anions and Acetazolamide

**DOI:** 10.1371/journal.pone.0028458

**Published:** 2011-12-05

**Authors:** Shenghua Huang, Tobias Hainzl, Christin Grundström, Cecilia Forsman, Göran Samuelsson, A. Elisabeth Sauer-Eriksson

**Affiliations:** 1 Department of Chemistry, Umeå University, Umeå, Sweden; 2 Department of Plant Physiology, Umeå Plant Science Centre, Umeå University, Umeå, Sweden; Griffith University, Australia

## Abstract

The β-class carbonic anhydrases (β-CAs) are widely distributed among lower eukaryotes, prokaryotes, archaea, and plants. Like all CAs, the β-enzymes catalyze an important physiological reaction, namely the interconversion between carbon dioxide and bicarbonate. In plants the enzyme plays an important role in carbon fixation and metabolism. To further explore the structure-function relationship of β-CA, we have determined the crystal structures of the photoautotroph unicellular green alga *Coccomyxa* β-CA in complex with five different inhibitors: acetazolamide, thiocyanate, azide, iodide, and phosphate ions. The tetrameric *Coccomyxa* β-CA structure is similar to other β-CAs but it has a 15 amino acid extension in the C-terminal end, which stabilizes the tetramer by strengthening the interface. Four of the five inhibitors bind in a manner similar to what is found in complexes with α-type CAs. Iodide ions, however, make contact to the zinc ion via a zinc-bound water molecule or hydroxide ion — a type of binding mode not previously observed in any CA. Binding of inhibitors to *Coccomyxa* β-CA is mediated by side-chain movements of the conserved residue Tyr-88, extending the width of the active site cavity with 1.5-1.8 Å. Structural analysis and comparisons with other α- and β-class members suggest a catalytic mechanism in which the movements of Tyr-88 are important for the CO_2_-HCO_3_^-^ interconversion, whereas a structurally conserved water molecule that bridges residues Tyr-88 and Gln-38, seems important for proton transfer, linking water molecules from the zinc-bound water to His-92 and buffer molecules.

## Introduction

Carbonic anhydrases (CAs, EC 4.2.1.1) are metalloenzymes, which catalyze the reversible hydration of carbon dioxide. CAs belong to five evolutionary distinct classes: α, β, γ, δ, and ζ which have no significant amino acid sequence identity and are thought to be the result of convergent evolution (for reviews see [1]–[4]). α-CA is the most extensively studied class and it is the only form found in vertebrates — there are more than ten isozymes of it identified in humans. CAs belonging to the β class have been mostly studied in plants but are found in all three kingdoms of life. The enzyme is present in plants with both C_3_ and C_4_ metabolism, in monocotyledons as well as dicotyledons, and in various photosynthesizing prokaryotes [1]. In higher plants, β-CA is localized to the chloroplast stroma of C_3_ plants, where it facilitates diffusion of CO_2_ across the stroma, and thus provides substrate for photosynthetic fixation by ribulose-1,5-biphosphate carboxylase (Rubisco) [5]. In plants with a C_4_ metabolism, β-CA is found in the cytosol of mesophyll cells, and is essential for converting CO_2_ to HCO_3_^-^, the substrate used by phosphoenolpyruvate carboxylase. The interpretation of the physiological function of CAs in microalgae is difficult due to the presence of multiple CA isozymes and different localizations. In microalgae that possess a carbon concentrating mechanism (CCM), the enzyme located in the chloroplast stroma is needed to convert the accumulated HCO_3_^-^ to CO_2_, the substrate for Rubisco [6].

*Coccomyxa* is a unicellular green alga that is mainly found in fresh water and soil, but it is also found growing in symbiosis in lichens where it acts as a photosynthetic component. *Coccomyxa* seems to lack a CCM [7]. The identified β-CA is located in the cytosol and interestingly the total CA activity of *Coccomyxa* is approximately 100 times higher than that of the CCM-containing alga *Chlamydomonas reinhardtii* [7], [8]. The specific function of β-CA in *Coccomyxa* (*Co*-CA) is still unclear. It is suggested that cytosolic β-CA facilitates diffusion of inorganic carbon from the inner surface of the plasmalemma to the chloroplast envelope [9], and that the high β-CA activity is correlated with a more efficient Rubisco in this alga compared with those that possess a CCM [10].

Three histidine residues coordinate the active site zinc ion in both α-CA and γ-CA. A fourth zinc ligand, a water molecule, ionizes to a hydroxide ion and actively participates in the catalytic event. Crystal structures have revealed two different zinc environments in β-CA, denoted type-I and type-II [4]. The crystal structure of *Pisum sativum* (pea) β-CA (*Ps*-CA) confirmed the Cys-His-Cys ligand scheme [11]. In the fourth position, an acetate ion was found, but it probably originated from the crystallization medium displacing the presumed water molecule. This structure represents the type-I (accessible) conformation of β-CA. In the X-ray structure of β-CA from the microalga *Porphyridium purpureum* (*Pp*-CA), the zinc was coordinated by a Cys-Asp-His-Cys tetrad without any water molecule within binding distance to the zinc [6]. With the zinc ion coordinated to four β-CA residues, this structure represents the type-II (blocked) conformation. So far structures of β-CA have also been determined from six other species [12]-[16]. In *Mycobacterium tuberculosis*, β-CA (*Mt*-CA) can switch between the two active-site conformations in a pH-dependent manner [13], [17]. At pH 7.0 the protein is dimeric and the active site is blocked, whereas at pH 7.5 the protein is tetrameric and the active site is accessible [13], [17]. Thus the blocked and accessible conformations in β-CAs seem to represent a catalytic on/off switch for the enzymes, coupled to pH and the oligomeric state [17].

Several of the CAs are very efficient enzymes, and the kinetic properties of human α-isozymeshuman CAI, II and III have been extensively investigated (see [18] for a review). With a *k*_cat_ value of 10^6 ^s^-1^, human CAII is one of the fastest enzymes known. Most β-CAs also have high catalytic efficiencies with *k*_cat_ values between 10^5^ and 10^6 ^s^-1^ and *k*_cat_/*K*_m_ values of 10^7^ - 10^8 ^M^−1^s^−1^ at high pH [4]. In the high-activity α-CA isozymes, the catalytically active entity is the zinc-bound water molecule, which ionizes to a hydroxide ion [18]-[23]. In the CO_2_-hydration reaction, the basic Zn-OH^-^ form of the enzyme is active, while the reverse reaction requires the protonated Zn-H_2_O form. The proposed mechanism divides the catalytic event into two stages. The first stage involves the interconversion of CO_2_ and HCO_3_^-^, whose rate is related to *k*_cat_/*K*_m_. The second stage is the regeneration of the active Zn-OH^-^ form of the enzyme, involving proton transfer from the zinc-bound water to the bulk buffer. In human CAII this transfer occurs in two steps, in which the amino acid His-64 shuttles the proton between the active site Zn-H_2_O and bulk buffer. At high buffer concentrations, the first intramolecular proton transfer step from the zinc-bound water to His-64 is rate limiting and the enzyme operates at its maximum. At low buffer concentrations the second proton transfer step from His-64 to the buffer molecule is rate limiting. Consequently, the second step is always reflected by the kinetic parameter *k*_cat_. The buffer thus participates in the reaction as a second substrate, resulting in a ping-pong mechanism [18].

Most of the kinetic properties observed for β-CAs are consistent with the mechanism for the α-CAs mentioned above. However, there are kinetic data that are not easily explained by that model, indicating that parts of the mechanism for β-CAs might differ from those of α-CAs. In human CAII, His-64 is positioned ∼7 Å from the zinc-bound water and the proton shuttle is mediated via four water molecules [24]. It has been suggested that two conserved residues, Tyr and His, play a similar role in β-CA proton transfer [25]. These residues are situated ∼6 Å and ∼10 Å respectively from the zinc-bound water. However, previous structural studies do not show how this proton transfer would be mediated.

In order to learn more about the molecular mechanism of β-CA, we have determined the crystal structure of β-CA from *Coccomyxa* (*Co*-CA) in complex with five inhibitors: acetazolamide, thiocyanate, azide, iodide, and phosphate ions. Previously, only thiocyanate-inhibited *Mt*-CA [17] and acetic acid-inhibited *Ps*-CA [11] have been reported. Our results support a zinc-hydroxide catalytic mechanism, similar to that of α-CA, in agreement with previous studies. Structurally conserved water molecules in the active site corroborate the involvement of Tyr-88 and His-92 in the proton transfer step.

## Results

### Description of overall structure

Crystals of *Co*-CA were obtained in the presence of high concentrations of phosphate ions (1.6–2.3 M). Phosphate ions bind to the active site zinc ions; thus we are currently lacking a native structure of the enzyme. Of the five inhibitor structures determined, the *Co*-CA-AZM structure was determined at the highest resolution, and refined at 1.9 Å (Table 1). Five residues at the N-terminal end of the enzyme (Met-1 to Asp-5) were not observed in the electron-density map, and these residues were omitted from the final models. Except for a few side-chains positioned at the surface of the protein, all residues are well defined in the electron density.

**Table 1:** Crystallization, data collection and refinement statistics.

	*Co*-CA-AZM	*Co*-CA-PO4	*Co*-CA-SCN	*Co*-CA-AZI	*Co*-CA-IOD
Condition of well solution	1.85 M NaKPO_4_20% glycerol	2.3 M NaKPO_4_20% glycerol	1.8 M NaKPO_4_20% glycerol	1.6 M NaKPO_4_	1.6 M NaKPO_4_
Concentration of inhibitor	10 µM AZM	–	15 mM NaSCN	2.5 mM NaN_3_	50 mM NaI
**Data collection**					
Data collected at	ESRF	ESRF	ESRF	In-house	In-house
Space group	P4_3_2_1_2	P4_3_2_1_2	P4_3_2_1_2	P4_3_2_1_2	P4_3_2_1_2
Cell dimensions (a, b, c):	74.56,74.56,220.39	74.06,74.06,220.50	76.84,76.84,222.14	75.65,75.65,222.22	75.48,75.48,222.41
wavelength(Å)	0.9311	0.9311	0.9311	1.5418	1.5418
Temperature (K)	100	100	100	293	293
range of resolution (Å)	20-1.85	20-2.50	20-2.50	20- 2.25	20-2.50
No. of observations	657320	220637	213006	138887	75678
No. of unique refl.	55540	22331	21341	29541	22929
completeness (%)	99.0	98.5	92.3	99.1	98.6
completeness (%) forthehighest resolution shell (Å)	98.6 (1.90-1.85)	100.0 (2.59-2.50)	98.2 (2.59-2.50)	97.2 (2.37-2.25)	99.7 (2.59-2.50)
R_sym_^a^	0.051/0.378	0.072/ 0.325	0.098/0.449	0.078/0.618	0.091/0.436
**Refinement:**					
No. of reflections	51036	20596	19246	29338	21690
R-factor^b^	0.181/0.281	0.205/0.260	0.189/0.270	0.160/0.270	0.153 /0.311
R_free_^c^	0.218/0.349	0.275/0.370	0.250/0.380	0.201/0.305	0.211 /0.370
Number of atoms:					
Protein	3404	3397	3390	3390	3390
Ligands	46	18	11	9	5
Water molecules	331	154	169	189	150
Overall B protein (Å^2^)	36.4	58.1	61.4	54.0	56.1
Overall B inhibitor (Å^2^)	35.3	58.9	56.6	44.0	68.3/30.4 (I^-^/H_2_O)
rmsdfor bonds (Å)	0.028	0.019	0.022	0.023	0.021
angles (°)	2.217	1.799	1.874	1.970	1.902

R_sym_^a^ for replicate reflections, R =  ΣI_hi_-<I_h_>|/Σ<I_h_>; I_hi_ = intensity measured for reflection h in data set i, <I_h_> = average intensity for reflection h calculated from replicate data.

R-factor^b^  =  Σ||Fo| - |Fc||/Σ|Fo|; Fo and Fc are the observed and calculated structure factors, respectively.

R_free_^ c^is based upon 10% of the data, randomly culled and not used in the refinement.

*Co*-CA is a homo-tetrameric structure with one dimer present in the asymmetric unit. Superposition of the two monomers in the asymmetric unit of *Co*-CA-AZM results in a root mean square (r.m.s.) deviation of 0.3 Å between corresponding α-carbon atoms (residues 6-227). The conformation of the five *Co*-CA complex structures is very similar. The only, and relatively minor, shift is found at the surface loop including residues Leu-141 to Leu-144, and with His-142 positioned at different conformations.

**Figure 1:**
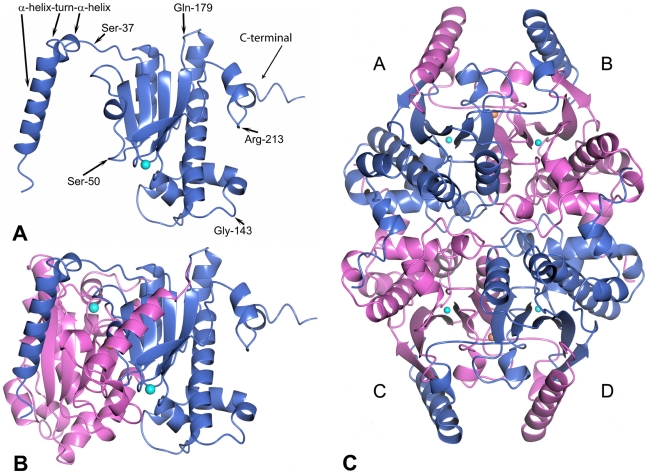
Ribbon drawings showing the fold and oligomerization of *Co*-CA. **A**: The monomer unit. The zinc ion is shown in cyan. For better orientation, the positions of some residues are indicated. **B**: The dimer unit. Contacts are mediated by strand β5, and helices α1 and α2 that wrap around the second monomer. **C**: The tetrameric structure. The tetramer is formed as a dimer of dimers. The chloride ions at the monomer-monomer interface are hidden in panel B, but visible in panel C as orange spheres.

Each monomer in *Co*-CA is built around a core comprising ten α-helices and five β-strands: four parallel β-strands (β2-β1-β3-β4), and one antiparallel β-strand (β5). The first 36 residues at the N-terminal end of each monomer form an α-helix-turn-α-helix motif in the shape of a bent arm (Fig. 1a). By swapping of these motifs, two monomers form a tight dimer (Fig. 1b). In all *Co*-CA-complex structures, anions bind at the monomer-monomer interface. In *Co*-CA-PO4, *Co*-CA-AZI and *Co*-CA-AZM, the anions were identified as chlorides present in the crystallization condition. For *Co*-CA-IOD and *Co*-CA-SCN, iodide and thiocyanate ions, respectively, substituted the chloride ions at the interface. The structure of the *Co*-CA tetramer is formed by applying a 2-fold crystallographic symmetry transformation on the dimer. The tetramer has 222 symmetry – a dimer of dimers – with approximate dimensions of 90×65×50 Å^3^ (Fig. 1c).

**Figure 2:**
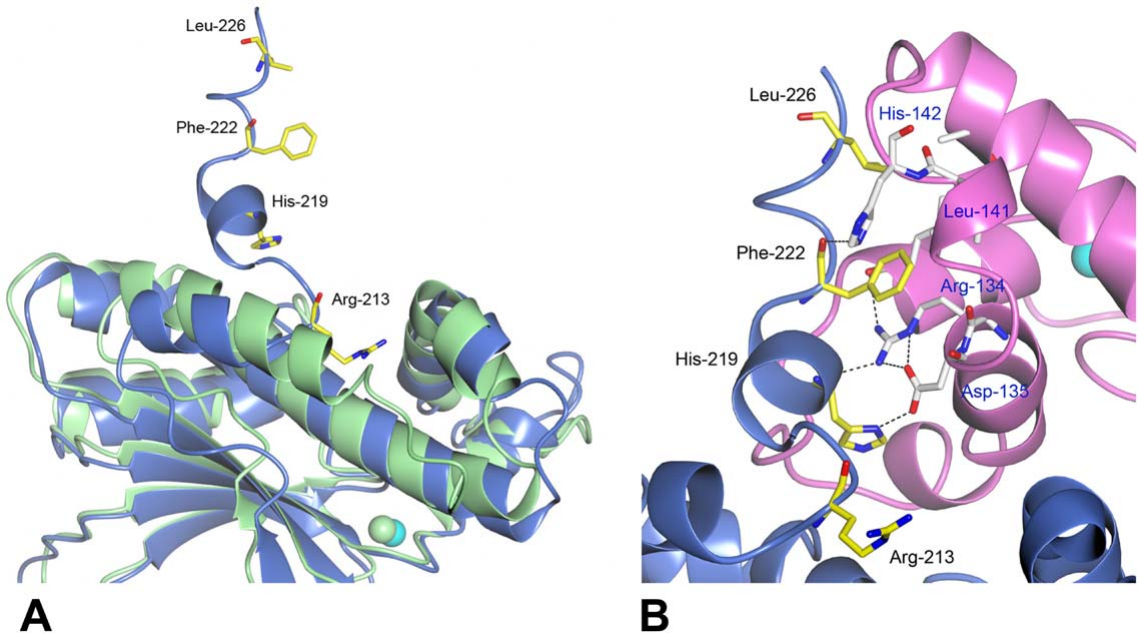
Interactions at the dimer-dimer interface involve residues at the C-terminal end of *Co*-CA. **A**: Superposition of *Co*-CA monomer A in blue on *Hi*-CA monomer E in green (pdb code 2A8D [14]). Whereas the C-terminal helix in *Hi*-CA extends over 4 turns, the helix in *Co*-CA breaks after two turns at residue Arg-213. **B**: The final 24 residues at the C-terminal in *Co*-CA are directed over the tetramer interface and make extensive hydrophobic- and hydrogen-bond contacts with a symmetry-related monomer (pink). Residues involved in dimer-dimer contacts are shown as ball-and-sticks. Bonds from carbon atoms in monomer A are colored in yellow, and bonds from carbon atoms in the symmetry-related monomer C are colored in white.

The central core of *Co*-CA resembles those of the other β-class CAs determined so far [6], [11]-[16]. Structural similarity searches using the DALI server [26], [27] identified CAs from the Gram-negative proteobacteria *E. coli* (*Ec*-CA) and *H. influenzae* (*Hi*-CA) as being most similar to *Co*-CA. Using secondary structure matching in PDBeFold [28] approximately 200 Cα atoms of *Co*-CA were aligned with *Hi*-CA and *Ec*-CA with an r.m.s. deviation of 1.2 Å and 1.6 Å, respectively, and sequence identities of 36–37%. The only major difference to previously determined structures is the shape of the extended C-terminal motif that contributes to the tetramer interface (Fig. 1). At residue Arg-213 in *Co*-CA, the C-terminal helix breaks and the remaining 14 residues are directed towards the dimer-dimer interface. In particular, the side-chains of His-119, Phe-222, Leu-226 are directed into the core of the second dimer across the tetramer interface to form hydrophobic contacts and hydrogen bonds to symmetry residues Leu-113, Ala-149, Arg-134, Asp-135, Leu-141, and His-142 (Fig. 2).

Another important residue at the tetramer interface is Lys-78. Its side chain is entirely buried in the second dimer and its Nζ atom forms three hydrogen bonds with the symmetry-related side chain of Asp-129, the main chain carbonyl oxygen of Trp-126, and with a buried water molecule. Generally, both the monomer-monomer and the dimer-dimer interfaces are polar and many of the interactions across the interfaces involve water molecules. Anions identified at the dimer interface are bound to the side chains of Arg-69 from both monomers and to water molecules.

There are nine cysteine residues in *Co*-CA including the two zinc-binding residues Cys-47 and Cys-106. None is involved in disulfide bridge formation.

**Figure 3:**
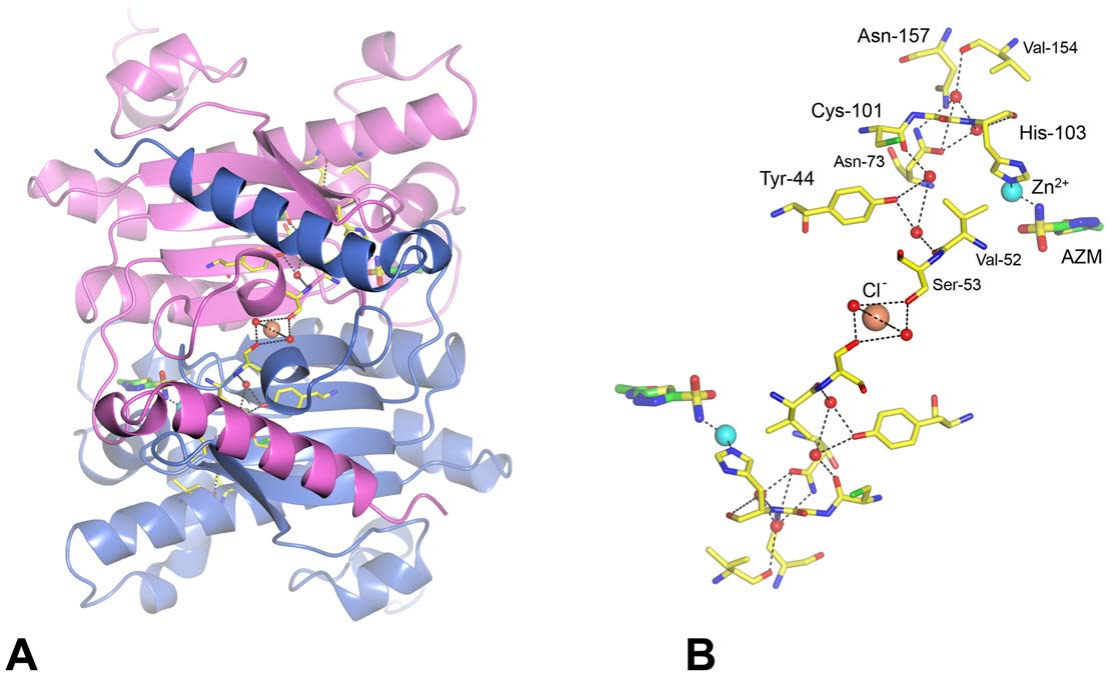
The buried hydrogen-bond network in *Co*-CA dimers. **A**: Ribbon representation. **B**: Ball-and-stick representation of the channel. Zinc and chloride ions are shown as cyan and orange spheres, respectively; water molecules are shown as red spheres. The buried four water molecules in each monomer are present in all five *Co*-CA structures.

331 bound water molecules were identified in the asymmetric unit of the 1.9 Å resolution structure of *Co*-CA-AZM. Of these, six are completely buried in the monomeric structure. In the dimer, ten water molecules together with several buried polar residues create an extended hydrogen-bonded network running through the interior of the protein (Fig. 3).

β-CAs can be inhibited by the same types of compounds as the α-CAs. Anionic inhibitors bind to the metal ion and prevent the formation of the zinc-coordinated OH^-^ ion, which is an essential participant in catalytic CO_2_ hydration. In this study, five inhibitors were co-crystallized with *Coccomyxa*β-CA and their structures analyzed.

### Acetazolamide binding

**Figure 4:**
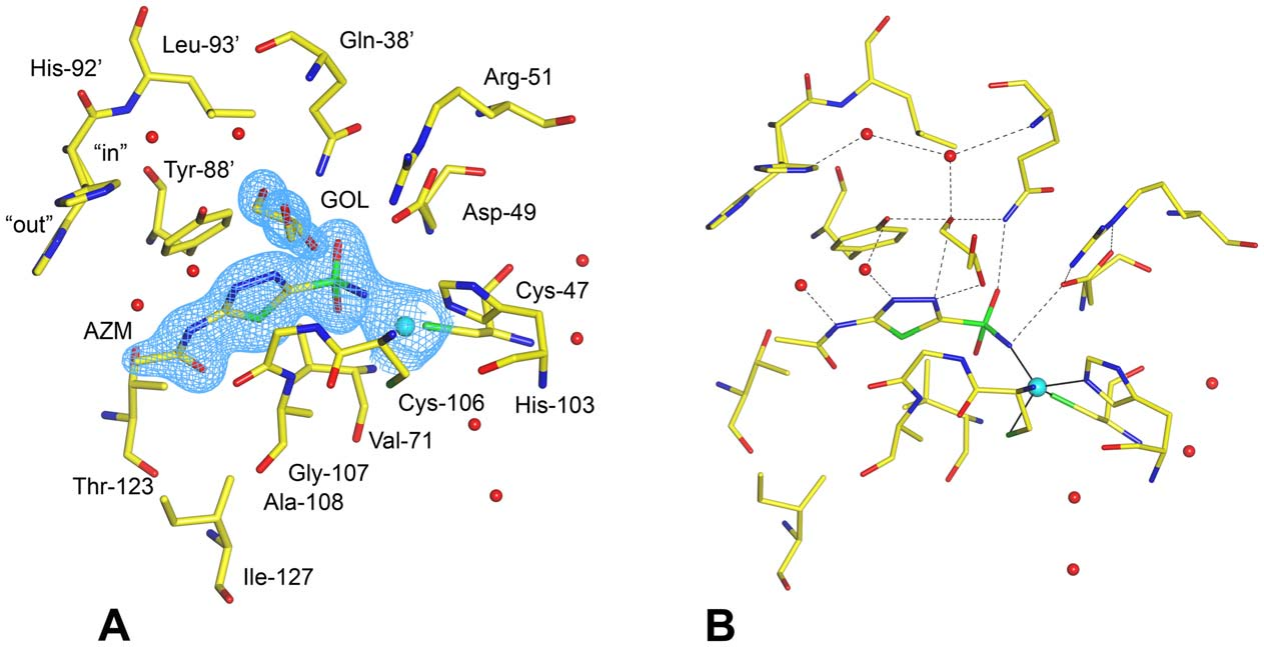
The active site of acetazolamide-inhibited *Co*-CA (*Co*-CA-AZM). **A**: 2|*F*_obs_|-|*F*_calc_| map of the catalytic site contoured at 1σ level. For clarity the map is calculated only over the acetazolamide, the glycerol molecule, and the zinc ion. Residues are shown in ball-and stick, zinc ion in cyan, and water molecules as red spheres. The glycerol molecule is labeled GOL. Primed residue numbers indicate symmetry-related residues in the dimer. **B**: Detailed view over the active site and the hydrogen-bonding network in *Co*-CA-AZM.

The structure of *Co*-CA in complex with acetazolamide, *Co*-CA-AZM, was refined at 1.9 Å. The maps show well-defined electron density for the entire acetazolamide molecule and for a neighboring glycerol molecule, both before and after refinement (Fig. 4a)**.** The active site cleft of *Co*-CA is located at the interface of the N-terminal surface of two monomers, and consists of a 10 Å deep and mostly hydrophobic cavity (Fig. 4b). The cavity has the shape of an hourglass where the narrowest part or "channel gate" is defined by Tyr-88′ on one side and Gly-107 and Ala-108 on the other. The wall of the channel is formed by residues, Asp-49, Arg-51, Gly-107, Ala-108, Ala-111, Val-114, and Trp-115 from one monomer, and Gln-38′, Phe-66′, Tyr-88′, His-92′, and Leu-93′ from the second monomer of the dimer. The prime symbol for residue numbers indicates that the residues belong to the symmetry*-*related monomer in the *Co*-CA dimer. The catalytic zinc ion binds to the side chains of residues Cys-47, His-103, and Cys-106 in a tetrahedral geometry with the fourth ligand expected to be a water molecule in the native enzyme. The zinc coordination and the geometry of the 14 residues in the active site of β-CA are conserved among CAs from bacteria, algae, and higher plants. A least-squares superposition of the 14 conserved residues of *Co*-CA and those of *Ps*-CA yields an r.m.s. deviation for Cα atoms of 0.5 Å.

The binding and orientation of AZM in *Co*-CA is similar to what has been observed in human CAII [29], and human CAI [30]. The amide group of the sulfonamide part of the inhibitor is situated 2.0 Å from the zinc atom and positioned in a tetrahedral geometry. Furthermore, the amide group forms a hydrogen bond to the Oδ2 atom of Asp-49 (3.1 Å). The O1 atom of the sulfonamide group forms a hydrogen bond to the Nδ2 atom of Gln-38′ (3.0 Å), whereas the last oxygen of the group forms no hydrogen bonds but is in van der Waals' contacts with Val-71 and Ala-108. The direction of the inhibitor is restricted by its thiadiazole ring, which is sandwiched at the "channel gate". The two nitrogen atoms in the thiadiazole ring form hydrogen bonds to a water and a glycerol molecule, respectively. The nitrogen of the N-acetamido group forms a hydrogen bond to a water molecule, whereas the acetamido group itself is buried in a rather hydrophobic part of the active site cleft.

### Phosphate ions

**Figure 5:**
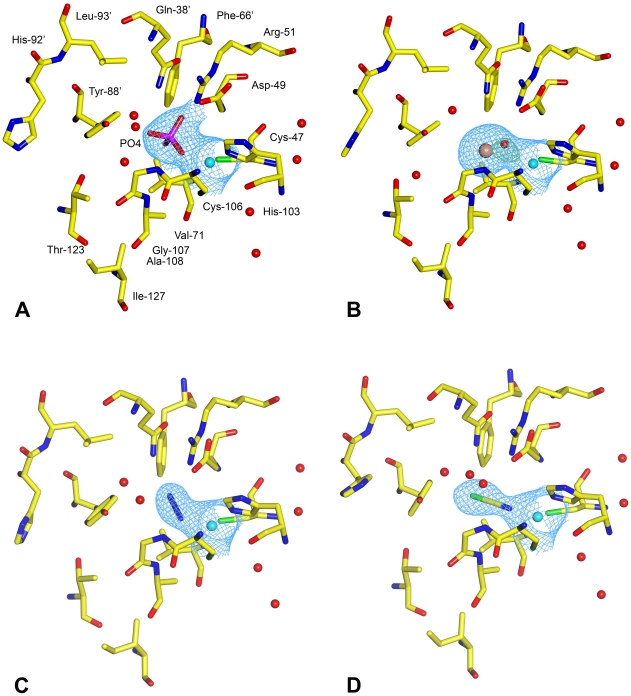
The active site of anion inhibited *Co*-CA. 2|*F*_obs_|-|*F*_calc_| maps of the catalytic site in *Co*-CA contoured at 1σ level. For clarity are all maps calculated only over the inhibitors and the zinc ions. **A**: *Co*-CA-PO4. **B**: *Co*-CA-IOD. The green mesh corresponds to the difference |*F*_obs_|-|*F*_calc_| map, calculated in the absence of the zinc-bound water molecule and contoured at 3σ level. **C**: *Co*-CA-AZI. **D**: *Co*-CA-SCN.

Crystals of *Co*-CA were obtained at high phosphate concentrations and studied at 2.5 Å resolution. An extraneous electron density feature appeared at the fourth ligand position of the catalytic zinc ion, indicated in the |*F*_obs_|-|*F*_calc_| map at 13σ and consistent with a bound phosphate ion (Fig. 5a). At pH 7.4 we assume HPO_4_^2-^ ions are binding to the zinc. However, we cannot exclude the possibility of occurrence of H_2_PO_4_^-^ ions. Binding of phosphate to the enzyme left the coordination geometry of the zinc ion tetrahedral with one phosphate oxygen atom (O1) situated 2.0 Å from the zinc ion, and forming a hydrogen bond (2.8 Å) with the main chain amide group of Gly-107. The three remaining oxygens of the phosphate are also involved in hydrogen bonds to surrounding residues in the active site: O2, which is probably protonated, interacts with the Oδ2 and Nδ2 atoms of Asp-49 and Gln-38′, respectively; O4 with the Oη group of Tyr-88′ and two water molecules; and O3, which points towards the hydrophobic pocket of Ala-48, Val-71, Phe-66′ and Tyr-88′, forms a hydrogen bond with the same water molecule as O4.

### Iodide binding

Binding of iodide ions to *Co*-CA was studied at 2.5 Å resolution. The |*F*_obs_|-|*F*_calc_| omit map showed a strong electron density peak at 35σ in the vicinity of the zinc. However, the center of the peak is positioned 3.9 Å from the zinc atom and not at 2.6–2.7 Å, which would have been the case if it bound directly to the zinc [31]. By comparison, the center of the HPO_4_^2-^ ion in the *Co*-CA-PO4 structure is situated 3.3 Å from the zinc. When modeled and refined as iodide, extra positive electron density at 6σ level remained between the positions of the zinc ion and the iodide ion. This density could only be interpreted as a water molecule, bridging the gap between the two ions (Fig. 5b). The zinc-bound water forms a hydrogen bond to the Oδ2 atom of Asp-49 (2.9 Å). The bond lengths from the water molecule to the iodide and zinc ions are 2.2 Å and 2.4 Å, respectively. In contrast to the phosphate ion, there are no additional water molecules surrounding the iodide; it is positioned within van der Waal contacts to Val-71, Ala-108, Phe-66′ and Tyr-88′. Partial refinement with both HPO_4_^2-^ and iodide ions present in the crystal lattice did not fit with electron density maps.

### Azide and thiocyanate binding

Azide and thiocyanate ions in the active site of *Co*-CA were clearly defined in the 2.3 Å and 2.5 Å electron density maps, respectively (Fig. 5c and 5d). The nitrogen atom (N1) of the azide ion binds to the zinc ion (2.0 Å) and forms a hydrogen bond to the amide group of Gly-107 (2.8 Å), while at the other end of the inhibitor, nitrogen (N3) forms a hydrogen-bond to the Nδ2 atom of Gln-38′ (3.3 Å). For SCN^-^ the nitrogen atom is also positioned 2.0 Å from the zinc ion, and makes a comparable, but weaker, hydrogen bond to the amide group of Gly-107 (3.4 Å). The orientation of the SCN^-^ ion was identified from the |*F*_obs_|-|*F*_calc_| omit map that showed a strong peak, above 10 σ, corresponding to the sulphur atom, and a weaker peak at 5σ, corresponding to the nitrogen atom. The geometry of the zinc is tetrahedral — the four substituent atoms binding to the zinc ion in *Co*-CA-SCN have bond angles between 100° and 120°. There is a water molecule bridging the Asp-49 Oδ2 atom and the NH atom of Gly-107 in the SCN^-^ inhibited structure.

### Structurally conserved water molecules in the active site of β-*Co*-CA

**Figure 6:**
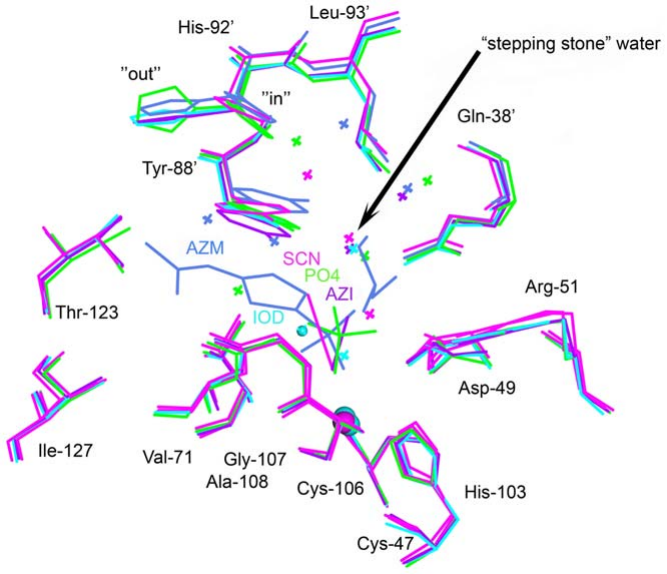
Superposition of anion- and sulfonamide-inhibited *Co*-CA. The color code is as follows: *Co*-CA-AZM, dark blue; *Co*-CA-PO4, green; *Co*-CA-IOD, cyan; *Co*-CA-AZI, dark purple; *Co*-CA-SCN, magenta. Zinc and iodide ions are shown as spheres, water molecules as crosses. Superpositions are based on the zinc-binding monomers only.

**Figure 7:**
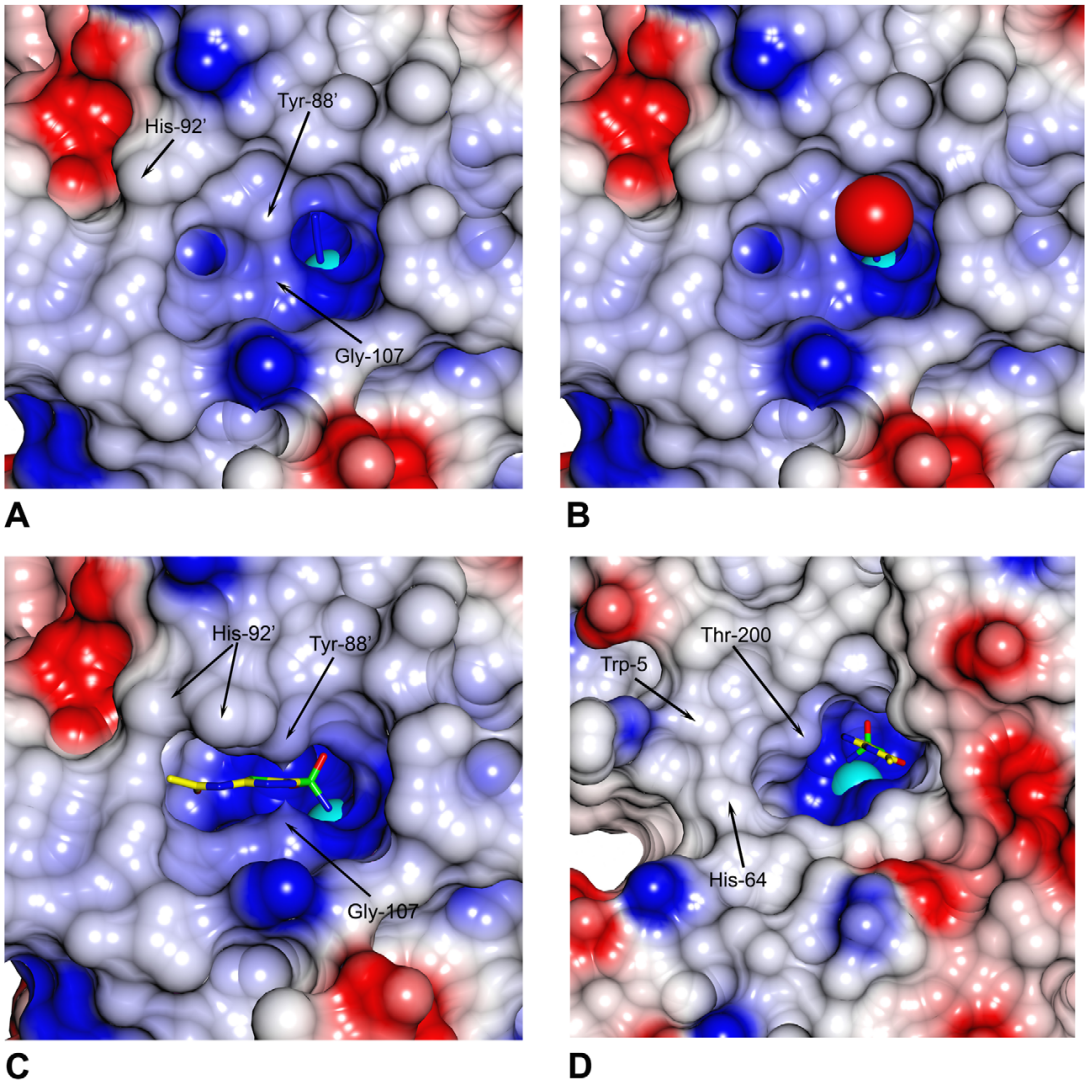
Accessibility and electrostatic potentials at the active sites of *Co*-CA and human CAII. Different CA enzymes have a differently shaped active site. The middle section i.e. the "channel gate" of the active site cavity in *Co*-CA is narrow. When the protein is in complex with the smallest inhibitors, i.e. azide ions, the "channel gate" seems closed due to the short distance between Tyr-88′, Gly-107 and Ala-108. **A**: *Co*-CA-AZI. **B**: *Co*-CA-AZI including the "stepping stone" water molecule. The water is shown as a red sphere with a radius of 1.2 Å. **C**: *Co*-CA-AZM with the acetazolamide shown as a ball-and-stick. **D**: The active site in the 1.1 Å structure of human CAII in complex with acetazolamide (pdb code 3HS4, [41]). The zinc ion is shown in cyan. Red and blue colors represent negative and positive potential, respectively. The elevated positive electrostatic potential at the active sites is due to the zinc ion included in the calculation.

Superposition of active site residues in the inhibitor-complex *Co*-CA structures revealed the positions of several structurally conserved water molecules. In particular, one water molecule is present in all structures except for *Co*-CA-AZM that has an oxygen from the glycerol molecule positioned at this site. The water molecule forms two hydrogen bonds: one to the Oη atom of Tyr-88′ and one to the Nε2 atom of Gln-38′. Both these residues are highly conserved within the β-CA family (Fig. 6). We refer to this water molecule as a "stepping stone" because of its strategic position effectively blocking the entrance to the active site cavity (Fig. 7a,b). Above the "stepping stone" water, additional water molecules are present making hydrogen bonds to the "stepping stone" water and either the main chain nitrogen atom of Gln-38′, or the Nε2 atom of His-92′. The presence of these water molecules is dependent on the side chain conformation of His-92′, the most flexible residue in the active site after Tyr-88′. The histidine side-chain has two conformations: an "in" conformation in *Co*-CA-SCN in which the side chain is directed towards the active site area, two conformations in *Co*-CA-AZM and *Co*-CA-PO4 (monomer B only, in monomer A it is "in"), and an "out" conformation in the structures of *Co*-CA-AZI, and *Co*-CA-IOD (Fig. 6). The water network linked to the “stepping stone” water is only present when His-92 is in its "in" conformation.

## Discussion

Lichens are composite organisms consisting of a symbiotic association of a fungus with a photosynthetic partner, usually a green alga or a cyanobacterium, or both. Lichens are extremophiles and are found in deserts, arctic tundra, and toxic wastelands [32]. Because most lichens are unable to regulate their water content they are completely dehydrated during long periods and their metabolic activity is neglectable. Surviving the dry season therefore requires certain adaptations on the molecular level, for example by increased protein stability. Lichens have the ability to take up water vapor from air, which stimulates CO_2_ assimilation so that high rates of photosynthesis are reached within a couple of hours in the absence of liquid water. *Coccomyxa* is a unicellular green alga that is common as a photosynthetic component of lichen. How living in harsh environments affects its proteins is not clear; however, *Coccomyxa* β-CA is significantly more stable and less sensitive to oxidation than its homologues in higher plants [8].

The five different classes of CAs known today diverge from one another with respect to their overall fold and quaternary structures [33]. α-CAs are mostly monomers whereas β-CAs form different types of oligomers. The *Coccomyxa* and bacterial CAs from e.g., *E. coli* form homotetramers [8], [34], whereas those from dicots like peas form homooctamers [35], and those from monocots form homodimers [36], [37]. β-CA from the alga *P. purpureum* contains two equivalent domains arranged in tandem, which combine with a second molecule to form a dimer with four active sites [6]. To learn more about the function of β-CA in plants and algae, we studied the structure of *Coccomyxa* β-CA in complex with inhibitors.

Previously determined structures of β-CAs are available from six different organisms including higher plants: *Pisum sativum* (pea) (*Ps*-CA, pdb code 1EKJ, [11]); microalgae *P. purpureum* (*Pp*-CA, pdb code 1DDZ, [6]); bacteria including *E. coli* (*Ec*-CA, pdb code 1I6P [12]), *Mycobacterium tuberculosis* (*Mt*-CA, pdb code 1YLK [13]) and *Haemophilus influenzae* (*Hi*-CA, pdb code 2A8D [14]); the chemoautotroph *Halothiobacillus neapolitanus* (pdb code 2FGY [15]), and the “cab”-type β-CA from the thermophilic archaeon *Methanobacteriumthermoautotrophicum* (pdb code 1G5C [16]). There is also one structure available from *Salmonella enterica* (pdb code 3QY1, unpublished). These structures fall into two distinct classes, called type I and II, classifications based on the organization of the active site region, in particular the ligation state of the active site zinc ion [4]. The structure of *Co*-CA described here is similar to previously studied structures, and shows the characteristics of a type I structure: (*i*) it has an exchangeable fourth ligand, (*ii*) an Asp-Arg dyad (residues Asp-49, Ser-50, Arg-51) that serves to orient the Asp residue to accept a hydrogen bond during catalysis, (*iii*) a hydrogen bond donor (residue Gln-38′), and finally (*iv*) a narrow hydrophobic active site cleft formed by residues Phe-66′, Val-71, Tyr-88′, His-92′ and Gly-107, which together form a continuous hydrophobic surface in the carbon dioxide binding pocket [4] (Fig. 4b).

**Figure 8:**
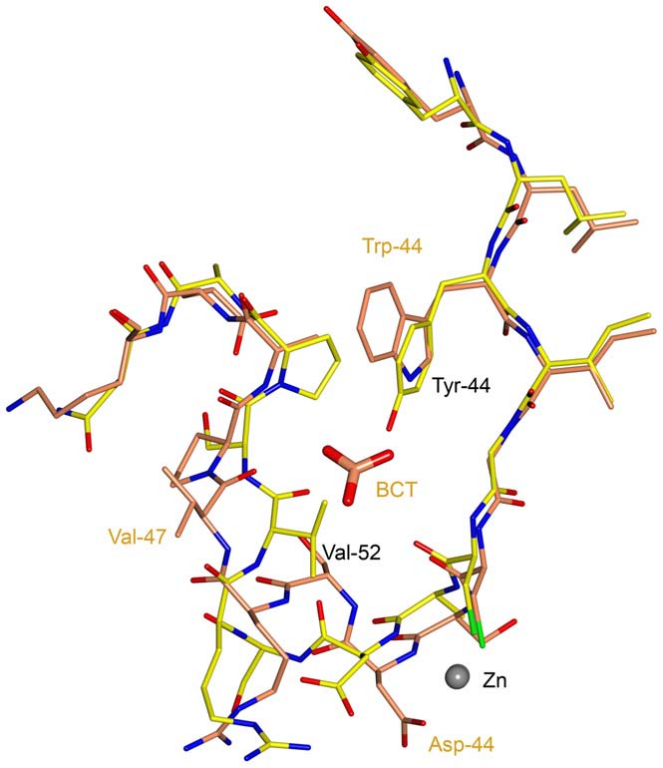
Superposition of the non-catalytic bicarbonate binding site in *Hi*-CA with the corresponding area in *Co*-CA. The close proximity of the Tyr-44 and Val-52 side chains prevents bicarbonate binding in *Co*-CA. The structure of *Co*-CA has carbon bonds colored in yellow, whereas the *Hi*-CA structure is shown with carbon bonds colored in orange (pdb code 2A8D, [14]). Superposition is based on residues 42-56 and 37-51 in *Co*-CA and *Hi*-CA, respectively.

Bicarbonate is the product of CA-catalyzed hydration of CO_2_. Structural and biochemical studies show that a non-catalytic binding site for HCO_3_^-^ exists in some, but not all members, of the β-CA family [14]. The binding site is present in *Hi*-CA and *Ec*-CA, and comprises residues Trp-39, Gly-41, Val-47, Arg-64 and Tyr-181, residues positioned in a pocket situated approximately 8 Å from the zinc ion [14]. Attempts to bind HCO_3_^-^to the *Co*-CA active site was complicated by the fact that a HPO_4_^2-^ ion bound to the zinc ion, and we could not elucidate if it was substituted with HCO_3_^-^ (data not shown). However, we did not detect any binding of HCO_3_^-^ at the presumed non-catalytic binding site in the co-crystals. In *Co*-CA, this site comprises residues Tyr-44, Gly-46, Val-52, Arg-69, and Tyr-188, and the loop structure that defines its shape is different compared to the bacterial structures (Fig. 8). Attempts to model HCO_3_^-^ at this site failed due to steric clashes. Therefore the non-catalytic HCO_3_^-^ binding site does not seem to occur in *Co*-CA.

Sulfonamides and anions are well-studied inhibitors of both α- and β-CAs [4], [18]. Acetazolamide binds stronger to the α-type CAs than to the β-type, whereas anions bind with approximately similar affinity to both types of CA [38]-[40]. Generally, anions and sulfonamides are more potent inhibitors of plant β-CA than of archaeal, bacterial or yeast β-CA. Acetazolamide is a better inhibitor of *Co*-CA than of β-CA from higher plants. The K_i_ of acetazolamide binding to *Co*-CA is 1 µM [8], which can be compared to the K_i_ values of acetazolamide binding to human CAII, human CAI, and pea CA with 0.01 µM, 0.2 µM, and 28 µM, respectively [38].

There are several structures known of α-CAs in complex with acetazolamide. These include human CAI, human CAII, *Neisseria gonorrhoeae* CA, human CAXII, and murine CAXIV [21], [29], [30], [41]–[43]. Generally, the NH group of the ionized sulfonamide group replaces the zinc-bound H_2_O/OH^-^ group, and its hydrogen atom forms a hydrogen bond to an acceptor residue on the protein. In this way, the inhibitor overcomes the “door-keeper” function of the acceptor residue, which tends to select protonated ligand atoms as replacements of the zinc-bound solvent molecule [44]. In human CAI and II, the “door-keeper” function is carried out by the Glu-106/Thr-199 system. In addition, one of the two sulfonamide oxygen atoms forms a hydrogen bond with the main chain amide group of Thr-199, whereas the second oxygen atom is situated approximately 3 Å from the zinc ion.

The binding mode of acetazolamide in the active site of *Co*-CA is similar to that seen in α-CA. One oxygen atom of the acetazolamide sulfonamide group forms a hydrogen bond to the amino group of Gln-38’ that acts as a hydrogen bond donor, in a manner similar to the main chain amide group of Thr-199 in α-CA. The ionized sulfonamide NH group coordinates to the zinc ion and donates a hydrogen bond to the Oδ2 atom of Asp-49, which has two electron lone pairs available for hydrogen bonding. The Nη2 atom of the guanidinium group of Arg-51 donates a hydrogen bond to the Asp-49 Oδ2 atom thereby orienting the second lone pair towards the zinc-bound H_2_O/OH^-^ group.

**Figure 9:**
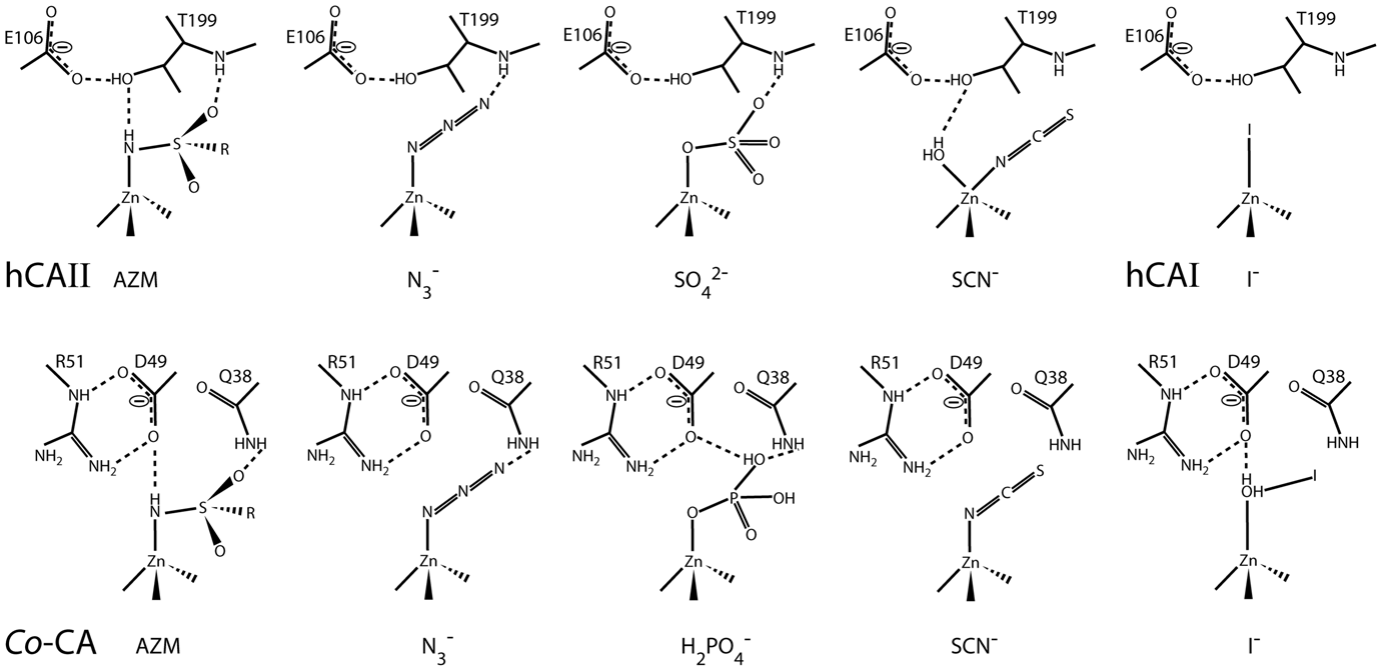
Schematic view illustrating the similarities of acetazolamide and anion binding in the structures of human CAII and *Co*-CA. For all structures the "door keeper" rule is fulfilled. The *Co*-CA representations show themirror-images of the active site to allow direct comparisons with the α-CA representations (adapted from [33]). hCAI and hCAII stand for human CA I and II, respectively.

The zinc-bound water molecule has been described in the active site of the "cab"-type β-CA from the archaeon *Methanobacterium thermoautotrophicum* [16]. Superposition of the conserved active site residues Cys-47, Asp-49, Arg-51, His-103, Cys-106, and zinc ion in the structure of *Co*-CA-AZM with the same residues in "cab", showed that the zinc-bound amide of the acetazolamide is present almost in the same position as the zinc-bound water molecule in "cab". It is likely that the hydroxide catalytic mechanism originates from this position in β-carbonic anhydrases. For the other four *Co*-CA inhibitor complexes the "door keeper" rule is also fulfilled (Table 2, Fig. 9). Furthermore, nitrogen and oxygen atoms of the azide and phosphate inhibitors, respectively, are hydrogen bonded to the amide group of Gln-38′. The iodide ion however, binds to the zinc ion via a bridging water molecule. This is different from the binding mode observed in α-human CAI, where the iodide is tetrahedrally coordinated directly to the zinc, replacing the zinc-bound water molecule [31]. Comparison of the two structures reveals differences in the active site and suggests a possible explanation for the difference in binding of the iodide ion to the two enzymes. In *Co*-CA, the presence of a negative charge, Asp-49 in the vicinity of the zinc ion, could prevent direct binding of the iodide ion to the zinc. In human CAII, however, iodide ions were reported to be positioned 3.9 Å from the zinc ion [45], and non-direct binding of iodide to the zinc ion agrees with the "door keeper" rule, in agreement with our finding. Further biochemical and biophysical studies of β-CA in complex with iodide are needed for the complete understanding of the mechanism behind iodide inhibition of *Co*-CA and other β-CAs. Thiocyanate binds together with a water molecule pentacoordinated to the zinc ion of α-human CAII [46]. In *Co*-CA, thiocyanate binds tetrahedrally to the zinc ion analogous to acetazolamide, azide and water (this work and [17]). The orientation of the thiocyanate ion in the *Mt*-CA structure [17] and in the *Co*-CA-SCN structure reported here is not identical. In *Mt*-CA, the sulphur atom of the inhibitor is positioned in the vicinity of Ala-75, i.e. the residue homologous to Val-71 in *Co*-CA. The sulphur atom in *Co*-CA-SCN is however directed towards Tyr-88′.

**Table 2:** The "door keeper" rule of inhibitor binding in *Co*-CA.

Inhibitor, atom	Zinc	Gly-107, NH	Asp-49, Oδ2
	(Distance (Å))	(Distance (Å))	(Distance (Å))
HPO_4_^2-^,O	2.0	2.8	3.6
N3^-^, N	2.0	2.8	3.8
SCN^-^, N	2.0	3.4	3.8
I^-^, OH (H_2_O)	2.3	3.4	2.9
AZM, NH	2.0	3.6	3.1

When zinc-binding groups are protonated, a hydrogen bond is formed to the side chain of the hydrogen acceptor Asp-49. If the zinc-bound group is not protonated, a hydrogen bond is formed to the main chain amide group of the hydrogen donator Gly-107.

Most of the kinetic properties observed for higher plant CAs are consistent with the catalytic mechanism for α-CAs. However, there are kinetic data that are not easily explained by the model, indicating that some parts of the mechanism for β-CA might differ from that of α-CA. If the ping-pong mechanism holds, then *k*_cat_/*K*_m_ would be unaffected by changing the solvent from H_2_O to D_2_O. This seems to be the case at high pH, but at pH 6 and 7, there is an isotope effect on *k*_cat_/*K*_m_ of 2.5 - 3, indicating that part of the mechanism may vary with pH [25], [47]. Further, high buffer concentrations are needed to obtain maximum activity for pea CA. In human CAII, His-64 functions as a proton acceptor/donator mediating rapid transport of protons between the zinc-bound water molecule and buffer molecules. On the basis of the kinetic data, it was suggested that two conserved residues, Tyr-88 and His-92 in *Co*-CA, (His-208 and Tyr-205 in pea β-CA, respectively), play the role of proton shuttle in β-CA [11]. Mutagenesis studies also support that these residues are involved in proton transport [25], [48]. Interestingly, we found that binding of inhibitors to *Co*-CA is mediated by side-chain movements of both residues Tyr-88′ and His-92′. For Tyr-88′ it is the Cα-Cβ-Oη angle of the side chain that is changing, whereas for His-92, movements involve rotation around the Chi-1 bond giving the residue two orientations — the "in" and the "out" (Fig. 6). In particular, the Tyr-88′′ movements can extend the width of the "channel gate" of the active site cavity with 1.5–1.8 Å (Table 3). Since the "channel gate" of β-CAs is very narrow in the small molecule inhibitor structures, changes in the Tyr-88′ conformation will affect the ability for diffusing molecules to pass in and out of the channel (Fig. 7a–c).

**Table 3:** The width of the "channel gate" of the active site cavity of β-CA.

β-CA structures	Tyr-88′ Oη -	Tyr-88′
	Gly-107 Cα	(Cα-Cβ-Oη)
	(Distance (Å))	(Angle (°))
*Co*-CA-AZM	5.68	107.8
*Co*-CA-SCN	4.55	111.8
*Co*-CA-PO4	4.50	111.2
*Co*-CA-IOD	4.44	111.6
*Co*-CA-AZI	3.84	116.3
*Pp*-CA	3.42	114.2
*Ps*-CA	3.43	113.6
*Hi*-CA	3.71	116.4
*Ec*-CA	4.10	114.7

The standard value for a Tyr (Cα-Cβ-Oη) angle is 113.95°.

**Figure 10:**
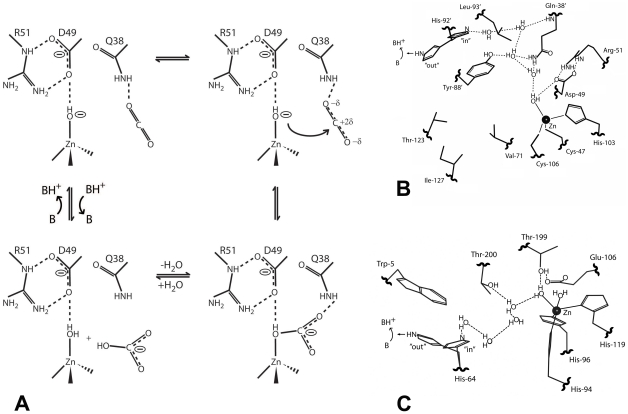
The catalytic mechanism of *Co*-CA. **A**: The CO_2_-HCO_3_^-^ interconversion step. **B**: The proposed mechanism for the proton transfer step in *Co*-CA. **C**: The proton transfer step in human CAII. The coordinates are from the 0.9 Å structure showing a penta-coordinated zinc atom (pdb code 3KS3 [56]).

The structures of *Co*-CA inhibitor complexes described here provide structural details for the catalytic mechanism of this class of enzymes. According to this mechanism, CO_2_ binds in the hydrophobic pocket with one oxygen caught by a hydrogen bond to the amide group of Gln-38′ (reviewed in [4]). The “door-keeper” role of the Asp-49/Arg-51 system orients the zinc-bound hydroxide ion so that its lone electron pairs make a nucleophilic attack on the carbon atom of the CO_2_ molecule. This is the first CO_2_-HCO_3_^-^ interconversion step of the mechanism and is probably mediated by movements of Tyr-88′ (Fig. 10a). The second step of the mechanism involves the regeneration of a hydroxide ion from the zinc-bound water molecule. However, Tyr-88′ and His-92′, suggested to play crucial roles in proton transport, are stacked on top of each other and are not aligned side-by-side, which would be necessary for a direct proton transfer from Tyr-88 to His-92, in a manner similar to, for example, residues Tyr-161 and His-190 in the D1 protein of photosystem II [49]. In human CAII, acetazolamide replaces the water network that bridges the zinc bound water molecule with His-64. In *Co*-CA, acetazolamide is positioned right at the "channel gate", i.e., at the narrowest part of the active site (Fig. 7c,d). For the small molecule inhibitor complexes the "channel gate" is normally closed. However, vertical to the direction of the long axis of acetazolamide molecule, there are structurally conserved water molecules that link residues Gln-38′, Tyr-88′ and His-92′ together (Fig. 6). In particular, one water molecule, referred to by us as the "stepping stone" water, is present in all of our *Co*-CA structures. These water molecules are also present in β-CA structures from other organisms, and we suggest that they play an active role in the proton transport step in a mechanism similar to that seen in human CAII (Fig. 10b,c). High activity α-CAs are monomeric proteins with structurally well-defined active sites, including a water network involved in proton transfer. Interestingly, in *Co*-CA as well as in other structures of β-CA, the His-92′ region of the proteins constitutes the most flexible part of the active site. His-92′ has an "in" and "out" conformation and the water structure varies between monomers with the exception of the "stepping stone" water molecule. In light of our current results this flexibility could reflect the functionality of the protein. β-CAs are oligomeric proteins and it is possible that not all active sites are functional at any one given time. Alternatively they are all functional but do not operate with the same catalytic efficiency. This is consistent with findings from a study that demonstrated intersubunit communication in cobalt-substituted HiCA dimer [50]. In such a scenario only the active sites with His-92′ in an "in" conformation and with an ordered water molecule network as outlined in figure 10b would be fully active.

## Materials and Methods

### Cloning, protein production and crystallization

The 227 amino acid β-CA from *Coccomyxa* sp. (*Co*-CA, accession number AAC33484.1) was overexpressed and purified as previously described [8]. Briefly, the protein was over-expressed in *E. coli* and purified on an amylose-resin column followed by a Q-Sepharose Fast Flow (GE Healthcare) ion-exchange column. For crystallization the protein was concentrated to ∼35 mg/ml in 10 mM Tris-HCl, pH 7.4. The crystals were grown at 18°C by the hanging-drop vapor-diffusion method. Initial hits were optimized by setting up droplets of 2-3 µl protein solution mixed with an equal volume of a reservoir solution in XRL plates (Molecular Dimensions). The largest and best diffracting crystals were obtained in 1.6-2.3 M Na,KPO_4_ pH 7.2 and grew to dimensions of 0.05×0.2×0.1 mm^3^ within two weeks. The X-ray structure revealed phosphate ions directly bound to the zinc ion, thus the structure represented a phosphate-inhibited complex of the enzyme (*Co*-CA-PO4). Other inhibitors were added to the protein solution for co-crystallization trials at the following concentrations: 2.5 mM NaN_3_ (*Co*-CA-AZI), 50 mM NaI (*Co*-CA-IOD), 10 µM acetazolamide (*Co*-CA-AZM), and 15 mM NaSCN (*Co*-CA-SCN).

### Data collection, structure solution and refinement

To prevent ice formation in the flash-cooled crystals used for X-ray data collection at 100 K, crystals had to be transferred to a cryoprotectant. We screened a large number of cryoprotecetants; however the crystals were fragile and difficult to handle. Diffraction data sets from crystals vitrified in ∼20% glycerol in Na,KPO_4_ could be collected for three of the inhibitor complexes:*Co*-CA-AZM, *Co*-CA-SCN, and *Co*-CA-PO4 at the European Synchrotron Radiation Facility, ESRF, in Grenoble, France. For the other two complexes, crystals were mounted in quartz capillary tubes and data were collected at 18°C on an in-house DIP2030H image plate detector, using CuKα radiation from a Nonius FR591 rotating anode operating at 49 kV and 90 mA.The space group was P4_3_2_1_2 and the data were processed and scaled using DENZO and SCALEPACK [51]. Statistics from data collections are listed in Table 1.

The *Co*-CA-AZM structure was solved by molecular replacement (MR) using the program CNS [52] and the *Ps*-CA structure, pdb code 1EKJ [11] asa search model. The electron density map was well defined and the model was built and refined using the programs Coot [53] and REFMAC5 [54]. The structures of the other four *Co*-CA-inhibitor complexes were determined by difference Fourier methods. Superposition of the structures was done in Coot using SSM [28]. Refinement statistics of the five structures are given in Table 1. Structural figures and electrostatic surfaces were prepared with CCP4 mg [55]. The atomic coordinates and structure factors (codes 3ucj (azm), 3uck (po4), 3ucm (scn), 3ucn (azi), and 3uco (iod)) have been deposited in the Protein Data Bank, Research Collaboratory for Structural Bioinformatics, Rutgers University, New Brunswick, NJ (http://www.rcsb.org/).

## References

[1] Hewett-Emmett D, Tashian RE (1996). Functional diversity, conservation, and convergence in the evolution of the alpha-, beta-, and gamma-carbonic anhydrase gene families.. Mol Phylogenet Evol.

[2] Ferry JG (2010). The gamma class of carbonic anhydrases..

[3] Supuran CT (2008). Carbonic anhydrases–an overview.. Curr Pharm Des.

[4] Rowlett RS (2010). Structure and catalytic mechanism of the beta-carbonic anhydrases..

[5] Badger MR, Price GD (2003). CO2 concentrating mechanisms in cyanobacteria: molecular components, their diversity and evolution.. J Exp Bot.

[6] Mitsuhashi S, Mizushima T, Yamashita E, Yamamoto M, Kumasaka T (2000). X-ray structure of beta-carbonic anhydrase from the red alga, Porphyridium purpureum, reveals a novel catalytic site for CO(2) hydration.. J Biol Chem.

[7] Hiltonen T, Karlsson J, Palmqvist K, Clarke AK, Samuelsson G (1995). Purification and characterisation of an intracellular carbonic anhydrase from the unicellular green alga Coccomyxa.. Planta.

[8] Hiltonen T, Bjorkbacka H, Forsman C, Clarke AK, Samuelsson G (1998). Intracellular beta-carbonic anhydrase of the unicellular green alga Coccomyxa. Cloning of the cdna and characterization of the functional enzyme overexpressed in Escherichia coli.. Plant Physiol.

[9] Badger MR, Price GD (1994). The role of carbonic anhydrase in photosynthesis.. Annu Rev Plant Physiol Plant Mol Biol.

[10] Palmqvist K, Sültemeyer D, Baldet P, Andrews TJ, Badger MR (1995). Characterisation of inorganic carbon fluxes, carbonic anhydrase(s) and ribulose-1,5-biphosphate carboxylate-oxygenase in the green unicellular alga Coccomyxa: comparison with low-CO_2_ cells of *Chlamydomonas reinhardtii.*. Planta.

[11] Kimber MS, Pai EF (2000). The active site architecture of Pisum sativum beta-carbonic anhydrase is a mirror image of that of alpha-carbonic anhydrases.. Embo J.

[12] Cronk JD, Endrizzi JA, Cronk MR, O'Neill J W, Zhang KY (2001). Crystal structure of E. coli beta-carbonic anhydrase, an enzyme with an unusual pH-dependent activity.. Protein Sci.

[13] Suarez Covarrubias A, Larsson AM, Hogbom M, Lindberg J, Bergfors T (2005). Structure and function of carbonic anhydrases from Mycobacterium tuberculosis.. J Biol Chem.

[14] Cronk JD, Rowlett RS, Zhang KY, Tu C, Endrizzi JA (2006). Identification of a novel noncatalytic bicarbonate binding site in eubacterial beta-carbonic anhydrase.. Biochemistry.

[15] Sawaya MR, Cannon GC, Heinhorst S, Tanaka S, Williams EB (2006). The structure of beta-carbonic anhydrase from the carboxysomal shell reveals a distinct subclass with one active site for the price of two.. J Biol Chem.

[16] Strop P, Smith KS, Iverson TM, Ferry JG, Rees DC (2001). Crystal structure of the "cab"-type beta class carbonic anhydrase from the archaeon Methanobacterium thermoautotrophicum.. J Biol Chem.

[17] Covarrubias AS, Bergfors T, Jones TA, Hogbom M (2006). Structural mechanics of the pH-dependent activity of beta-carbonic anhydrase from Mycobacterium tuberculosis.. J Biol Chem.

[18] Lindskog S (1997). Structure and mechanism of carbonic anhydrase.. Pharmacol Ther.

[19] Alberti G, Bertini I, Luchinat C, Scozzafava A (1981). A new class of inhibitors capable of binding both the acidic and alkaline forms of carbonic anhydrase.. Biochim Biophys Acta.

[20] Tibell L, Forsman C, Simonsson I, Lindskog S (1984). Anion inhibition of CO2 hydration catalyzed by human carbonic anhydrase II. Mechanistic implications.. Biochim Biophys Acta.

[21] Huang S, Xue Y, Sauer-Eriksson E, Chirica L, Lindskog S (1998). Crystal structure of carbonic anhydrase from Neisseria gonorrhoeae and its complex with the inhibitor acetazolamide.. J Mol Biol.

[22] Supuran CT (2008). Diuretics: from classical carbonic anhydrase inhibitors to novel applications of the sulfonamides.. Curr Pharm Des.

[23] Lindskog S, Silverman DN (2000). The catalytic mechanism of mammalian carbonic anhydrases.. Exs.

[24] Eriksson AE, Jones TA, Liljas A (1988). Refined structure of human carbonic anhydrase II at 2.0 A resolution.. Proteins.

[25] Rowlett RS, Tu C, McKay MM, Preiss JR, Loomis RJ (2002). Kinetic characterization of wild-type and proton transfer-impaired variants of beta-carbonic anhydrase from Arabidopsis thaliana.. Arch Biochem Biophys.

[26] Holm L, Sander C (1993). Protein structure comparison by alignment of distance matrices.. J Mol Biol.

[27] Holm L, Kaariainen S, Rosenstrom P, Schenkel A (2008). Searching protein structure databases with DaliLite v.3.. Bioinformatics.

[28] Krissinel E, Henrick K (2004). Secondary-structure matching (SSM), a new tool for fast protein structure alignment in three dimensions.. Acta Crystallogr D Biol Crystallogr.

[29] Vidgren J, Liljas A, Walker NP (1990). Refined structure of the acetazolamide complex of human carbonic anhydrase II at 1.9 A. Int J Biol Macromol.

[30] Chakravarty S, Kannan KK (1994). Drug-protein interactions. Refined structures of three sulfonamide drug complexes of human carbonic anhydrase I enzyme.. J Mol Biol.

[31] Kumar V, Kannan KK, Sathyamurthi P (1994). Differences in anionic inhibition of human carbonic anhydrase I revealed from the structures of iodide and gold cyanide inhibitor complexes.. Acta Crystallogr D Biol Crystallogr.

[32] Nash TH (2008). Lichen Biology, 2nd Ed., Cambridge, University Press, NY.

[33] Liljas A, Laurberg M (2000). A wheel invented three times. The molecular structures of the three carbonic anhydrases.. EMBO Rep.

[34] Guilloton MB, Korte JJ, Lamblin AF, Fuchs JA, Anderson PM (1992). Carbonic anhydrase in Escherichia coli. A product of the cyn operon.. J Biol Chem.

[35] Bjorkbacka H, Johansson IM, Skarfstad E, Forsman C (1997). The sulfhydryl groups of Cys 269 and Cys 272 are critical for the oligomeric state of chloroplast carbonic anhydrase from Pisum sativum.. Biochemistry.

[36] Atkins CA, Patterson BD, Graham D (1972). Plant Carbonic Anhydrases: I. Distribution of Types among Species.. Plant Physiol.

[37] Atkins CA, Patterson BD, Graham D (1972). Plant Carbonic Anhydrases: II. Preparation and Some Properties of Monocotyledon and Dicotyledon Enzyme Types.. Plant Physiol.

[38] Johansson IM, Forsman C (1993). Kinetic studies of pea carbonic anhydrase.. Eur J Biochem.

[39] Rowlett RS, Chance MR, Wirt MD, Sidelinger DE, Royal JR (1994). Kinetic and structural characterization of spinach carbonic anhydrase.. Biochemistry.

[40] Pacchiano F, Carta F, Vullo D, Scozzafava A, Supuran CT (2011). Inhibition of beta-carbonic anhydrases with ureido-substituted benzenesulfonamides.. Bioorg Med Chem Lett.

[41] Sippel KH, Robbins AH, Domsic J, Genis C, Agbandje-McKenna M (2009). High-resolution structure of human carbonic anhydrase II complexed with acetazolamide reveals insights into inhibitor drug design.. Acta Crystallogr Sect F Struct Biol Cryst Commun.

[42] Di Fiore A, Monti SM, Hilvo M, Parkkila S, Romano V (2009). Crystal structure of human carbonic anhydrase XIII and its complex with the inhibitor acetazolamide.. Proteins.

[43] Whittington DA, Grubb JH, Waheed A, Shah GN, Sly WS (2004). Expression, assay, and structure of the extracellular domain of murine carbonic anhydrase XIV: implications for selective inhibition of membrane-associated isozymes.. J Biol Chem.

[44] Liljas A, Hakansson K, Jonsson BH, Xue Y (1994). Inhibition and catalysis of carbonic anhydrase. Recent crystallographic analyses.. Eur J Biochem.

[45] Liljas A, Kannan KK, Bergsten PC, Waara I, Fridborg K (1972). Crystal structure of human carbonic anhydrase C. Nat New Biol.

[46] Eriksson AE, Kylsten PM, Jones TA, Liljas A (1988). Crystallographic studies of inhibitor binding sites in human carbonic anhydrase II: a pentacoordinated binding of the SCN- ion to the zinc at high pH.. Proteins.

[47] Johansson IM, Forsman C (1994). Solvent hydrogen isotope effects and anion inhibition of CO2 hydration catalysed by carbonic anhydrase from Pisum sativum.. Eur J Biochem.

[48] Bjorkbacka H, Johansson IM, Forsman C (1999). Possible roles for His 208 in the active-site region of chloroplast carbonic anhydrase from Pisum sativum.. Arch Biochem Biophys.

[49] Umena Y, Kawakami K, Shen JR, Kamiya N (2011). Crystal structure of oxygen-evolving photosystem II at a resolution of 1.9 A. Nature.

[50] Hoffmann KM, Samardzic D, Heever K, Rowlett RS (2011). Co(II)-substituted Haemophilus influenzae beta-carbonic anhydrase: Spectral evidence for allosteric regulation by pH and bicarbonate ion.. Arch Biochem Biophys.

[51] Otwinowski Z, Borek D, Majewski W, Minor W (2003). Multiparametric scaling of diffraction intensities.. Acta Crystallogr A.

[52] Brunger AT, Adams PD, Clore GM, DeLano WL, Gros P (1998). Crystallography & NMR system: A new software suite for macromolecular structure determination.. Acta Crystallogr D Biol Crystallogr.

[53] Emsley P, Cowtan K (2004). Coot: model-building tools for molecular graphics.. Acta Crystallogr D Biol Crystallogr.

[54] Murshudov GN, Vagin AA, Dodson EJ (1997). Refinement of macromolecular structures by the maximum-likelihood method.. Acta Crystallogr D Biol Crystallogr.

[55] Potterton E, McNicholas S, Krissinel E, Cowtan K, Noble M (2002). The CCP4 molecular-graphics project.. Acta Crystallogr D Biol Crystallogr.

[56] Avvaru BS, Kim CU, Sippel KH, Gruner SM, Agbandje-McKenna M (2010). A short, strong hydrogen bond in the active site of human carbonic anhydrase II.. Biochemistry.

